# Open Science at an institutional level: an interview with Guy Rouleau

**DOI:** 10.1186/s13059-017-1152-z

**Published:** 2017-01-20

**Authors:** Guy Rouleau

**Affiliations:** 1 Montreal Neurological Institute, Montreal, QC H3A 2B4 Canada; 2 Department of Neurology and Neurosurgery, McGill University, Montreal, QC H3A 2B4 Canada

## Abstract

The director of the Montreal Neurological Institute (MNI), Guy Rouleau, discusses the recent announcement that the MNI will be completely committed to open science.

## You recently announced that the Montreal Neurological Institute is about to become a fully open data institute. What does this entail?

Treatments for the diseases of the brain and nervous system are one of the most compelling unmet medical needs of our time. At the MNI, we see the most complex of neurological and neurosurgical cases. For many of these patients, we have not been able to offer any new treatments for decades. The technology available to us today can enable the scientific community to share information and speed up discoveries. Seeing these patients and knowing the talented researchers we have, it makes sense to share as much information as possible.

In establishing itself as the world’s first academic institution operating fully on the principles of Open Science, the MNI is realizing a vision in which early-stage drug research is open, borderless, and global; world-class institutions around the world share expertise and collaborate on fundamental discoveries; and new treatments and even cures for devastating neurological diseases are within this generation’s reach, promising enormous human and economic benefits for the healthcare system and economy.

To achieve these goals, Open Science at the MNI will be driven along five key axes: Open Access, Open Data, Open Intellectual Property (IP), Open Biobank, and Open Commercialization. That is, publishing research results without restriction; sharing experimental data freely with institutions around the world; refraining from pursuing patents on MNI-generated discoveries; freely sharing biological samples and other resources from the MNI Biobank (C-BIGR; see the answer to Question 6, below), within the limits of supply and respecting patient confidentiality; and developing business models to bring open-source discoveries, including new medicines, to the marketplace.

## What do you think the benefits will be of this step?

From its inception, The MNI’s singular mission has been to deploy scientific research in the service of patients, families, and society. We see Open Science as a means to expand the impact of our research by sharing it with a global community of like-minded scientists.

We hope that the benefits will include a better understanding of the mechanisms of neurological diseases, new therapeutic targets, and ultimately new treatments for our patients. We believe that increasing openness in research and operating along principles of collaboration will improve transparency, reliability, and the impact of research to accelerate the pace of discoveries that could help patients around the world who have devastating neurological diseases and disorders. By allowing researchers from around the world with many different interests and expertise to access our data, research will progress exponentially for the benefit of patients.

The MNI will also set a precedent for other research institutes in adopting the principles of Open Science, leading in the development of new policies and tools required to drive a faster and more complete exchange of scientific knowledge. The hope is that we can share our experience with the wider scientific community and serve as a case study and blueprint that other scientific institutions can follow in adopting the principles of Open Science.

In the last two years, we have hired more than a dozen new researchers and physicians; we are in the midst of a great rejuvenation. We predict that our new Open Science policy will increase—and already has increased—our capacity to attract highly talented researchers and trainees who are highly committed to and engaged in the Open Science philosophy.

## How long has it taken you to get to the point from first discussing the idea to being ready to commit to being open?

The MNI has a long history of Open Science, particularly in brain imaging. We have one of the three largest brain-imaging centers in the world. Researchers around the globe use the Atlas of the Human Brain in Stereotaxic Space (http://www.thehumanbrain.info/brain/index.php), and our scientists have developed software and image-analysis platforms that are open access and set a gold standard.

Personally, as a physician and clinician-researcher, I have always been convinced that Open Science is the right thing to do ethically. On the basis of that personal conviction, I have decided to persuade the rest of the community to take this step. Around 18 months passed between the first discussions of the idea to committing to being open.

## The faculty vote was passed unanimously, but were there faculty members who were reluctant and needed to be persuaded?

I would say that because of this tradition at MNI, none of our faculty were reluctant, but some raised the very relevant and important point of feasibility and questioned the potential additional work burden that this new initiative would create on their own activities.

We worked on a buy-in process that started by defining an MNI-tailored Open Science approach and framework and key elements to consider. We then mapped existing open science activities versus closed activities at the MNI. For over a year, we engaged in a consultation process with MNI faculty, staff, and students, consisting of seminars, polls, town hall meetings, and Q&A sessions. An independent research group was commissioned to perform a social science study using structured questionnaires to identify potential barriers and limitations related to Open Science. Finally, we established Guiding Principles with the agreement of all MNI members.

## The institute is not pursuing IP. Universities often see patents as a useful source of income: did you receive any resistance from the finance department at McGill over your stand on IP?

Since taking the very first steps towards our vision of becoming an Open Science organization, we had very strong and powerful support from McGill University’s Principal, Suzanne Fortier, and many other senior members of the University, confirming that our approach was consistent with the university’s intellectual property policy that allows researchers to decide whether to publish or commercialize their research and inventions.

On the basis of the MNI experience with Open Science, there is a clear potential to spread these principles and their benefits more broadly at the university level as well as at a global level.

Furthermore, the kind of science that the MNI does is so early-stage that we would rather provide data that others could use to develop patentable medicines. It comes back to what we aim for: accelerating science, not making money.

Of course there is a risk that we might lose the economic returns of a blockbuster drug or a new intervention, but we are ethically committed to taking that risk, as the bigger risk is for our patients who are waiting for answers and new treatments.

## As a neurological institute, openness requires consent from the patients that you see. Has this been a problem?

The objective of our Clinical, Biological, Imaging and Genetic data Repository (C-BIGR) is to collect biological material as well as clinical and imaging information from patients and research participants in order to enable cutting-edge research projects that will advance our understanding of neurological diseases. Aggregating collections of material and data from patients who have neurological diseases, some of which are rare, will permit investigations into the fundamental molecular alterations underlying patient disease; permit discovery of biomarkers to improve patient diagnosis; and permit tissue-based translational research that will allow us to develop new treatments that can alter the natural history of these devastating diseases.

The C-BIGR will also empower patients by allowing them to participate indirectly in research aimed at advancing our understanding of their diseases.

Our patients perceive this initiative as extremely important and crucial and are highly supportive, not only for themselves but mainly for the generations to come that would benefit from the extraordinary outcomes of such a culture and new way of doing research.

Over 90% of patients agree to participate.

## What do you think are going to be the biggest challenges of being completely open?

The first challenge is to be able to make meaningful data accessible, not just raw data. The second challenge will certainly be building the right infrastructure to support our community in achieving this. A third challenge that we foresee is the ability to deploy mechanisms to measure, increase the impact of, and fully acknowledge our researchers’ and trainees’ contributions in the Open Science context. A fourth and obvious challenge is the shift of mindset implied by this new policy that will allow people to work more collectively in a community, both at an individual level for each researcher in their own lab and at an institutional level.

## Is the institute now as open as it can be, or are there levels of openness that currently can’t be reached because of legal or technical problems?

We are currently working on deploying the full suite of tools and platforms that constitute the support infrastructure for Open Science. Our Open Science policy and its guiding principles are now integrated into all contract agreements with our partners, both public and private.

On the legal side, issues regarding third parties’ existing intellectual property rights on techniques or methods used by our researchers in their labs—for instance, the existing international legal battle on CRISPR technology—may actually represent legal limitations or hurdles to the confirmation of new partnerships with industry.

